# Temozolomide promotes matrix metalloproteinase 9 expression through p38 MAPK and JNK pathways in glioblastoma cells

**DOI:** 10.1038/s41598-024-65398-2

**Published:** 2024-06-21

**Authors:** Hien Duong Thanh, Sueun Lee, Thuy Thi Nguyen, Thang Nguyen Huu, Eun-Jung Ahn, Sang-Hee Cho, Min Soo Kim, Kyung-Sub Moon, Chaeyong Jung

**Affiliations:** 1 Department of Anatomy, Chonnam National University Medical School, Gwangju, 61469 Korea; 2 Herbal Medicine Resources Research Center, Korea Institute of Oriental Medicine, Naju-Si, 58245 Jeollanam-Do Korea; 3 Department of Biochemistry, Chonnam National University Medical School, Gwangju, 61469 Korea; 4 Department of Neurosurgery, Chonnam National University Hwasun Hospital and Medical School, Hwasun, 58128 Jeollanam-Do Korea; 5 Department of Hemato-Oncology, Chonnam National University Medical School, Gwangju, 61469 Korea; 6 Department of Statistics, College of Natural Sciences, Chonnam National University, Gwangju, 61186 Korea

**Keywords:** Cell signalling, Cancer

## Abstract

Glioblastoma (GBM) is a highly aggressive and deadly brain cancer. Temozolomide (TMZ) is the standard chemotherapeutic agent for GBM, but the majority of patients experience recurrence and invasion of tumor cells. We investigated whether TMZ treatment of GBM cells regulates matrix metalloproteinases (MMPs), which have the main function to promote tumor cell invasion. TMZ effectively killed GL261, U343, and U87MG cells at a concentration of 500 µM, and surviving cells upregulated MMP9 expression and its activity but not those of MMP2. TMZ also elevated levels of *MMP9* mRNA and *MMP9* promoter activity. Subcutaneous graft tumors survived from TMZ treatment also exhibited increased expression of MMP9 and enhanced gelatinolytic activity. TMZ-mediated MMP9 upregulation was specifically mediated through the phosphorylation of p38 and JNK. This then stimulates AP-1 activity through the upregulation of c-Fos and c-Jun. Inhibition of the p38, JNK, or both pathways counteracted the TMZ-induced upregulation of MMP9 and AP-1. This study proposes a potential adverse effect of TMZ treatment for GBM: upregulation of MMP9 expression potentially associated with increased invasion and poor prognosis. This study also provides valuable insights into the molecular mechanisms by which TMZ treatment leads to increased MMP9 expression in GBM cells.

## Introduction

Glioblastoma (GBM) is a very aggressive cancer that accounts for most primary brain tumor cases^1^. It is classified as the most malignant grade IV glioma. The clinical prognosis is poor despite several approved therapies for GBM, including surgery, radiation, and chemotherapy^1^. Temozolomide (TMZ; commercially known as Temodar) is a DNA alkylating agent and widely used because it is the most effective drug in the current standard therapeutic protocol for glioblastoma. The therapeutic potential of TMZ has been proven in treating primary and relapsed glioblastomas, increasing the overall survival of glioblastoma patients. However, significant numbers of recurrences and drug resistance have been observed^2^. The 5-year survival rate of GBM patients is only 4–5%, and the median survival is 14–15 months due to the recurrence of cancer cells^3^. These cells can escape current therapeutic treatments and become resistant to drugs whose actions are mediated by the DNA repair protein, O6-methyguanine-DNA methyltransferase (MGMT)^4^. Poor prognoses are also because of the diffusely invasive and heterogenous nature of GBM tumor cells, which makes complete resection nearly impossible even after total resection of a brain hemisphere^5^.

The mitogen-activated protein kinase (MAPK) pathways play essential roles in transducing extracellular signals for cellular responses, including inflammation, differentiation, proliferation, apoptosis, and cell survival^6^. The main members of the MAPK family are extracellular signal-regulated kinases (ERK), p38, and c-Jun N-terminal kinases (JNK), all of which respond to cytokines, bacteria, and physical and chemical stresses^7^. Activator protein-1 (AP-1) is a dimeric complex mainly assembled and synthesized by Jun and Fos family proteins, which are affected by MAPK pathway activity^7–9^. Matrix metalloproteinases (MMPs) are enzymes in the zinc-endopeptidase family that participate in extracellular matrix (ECM) breakdown^10,11^. MMPs are secreted by the cell into the extracellular environment or bound to the plasma membrane. Depending on substrate specificity, sequence similarity, and domain organization, MMPs, including MMP2 and MMP9, are classified into several groups^12^. Under normal physiological conditions, the principal biological functions of MMPs play essential roles in angiogenesis, tissue repair and remodeling, wound healing, cell proliferation, morphogenesis, mobility, invasion, and cell migration. MMPs are tightly controlled at several levels, such as at the transcriptional and post-transcriptional levels and by enzyme activation, specific inhibitors, and enzyme degradation^13^. The dysregulation of MMPs expression and activity leads to various diseases, including cancer^10^. MMP9 is a gelatinase that degrades type 4 collagen in the basement membrane of cells, creating a microenvironment where cancer cells can invade and metastasize^10,14^. MMP9 is overexpressed in various cancers, including in glioblastoma, and its overexpression in GBM is associated with a poor prognosis due to tumor progression and cancer recurrence^15–19^. However, there is limited information about the possible relationship between TMZ treatment of GBM cells and MMP9 expression and activity^20,21^. This study investigated the effects of TMZ in GBM on MMP9 and the mechanism underlying how MMP9 is affected by TMZ.

## Results

### TMZ suppresses GBM cell growth and adversely upregulates MMP9 expression

**Figure 1:**
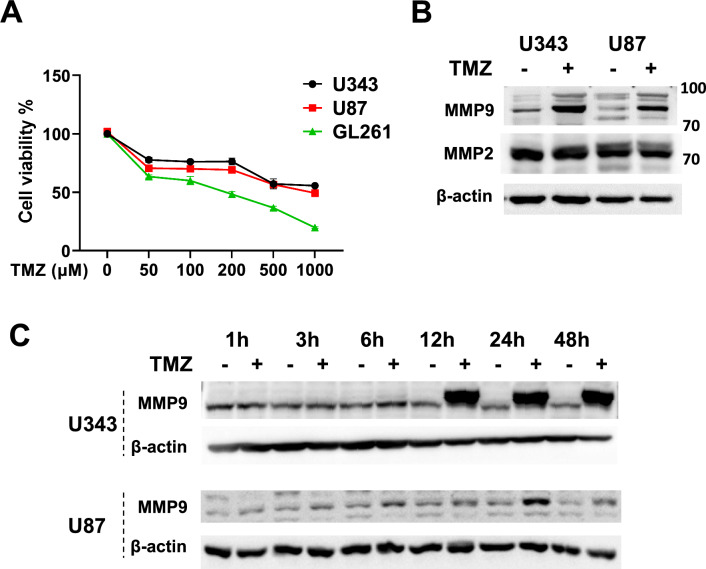
Effects of TMZ on the expression of MMP9 in GBM cells. An MTT cell proliferation assay was used to measure TMZ cytotoxicity. Cells were incubated with TMZ at concentrations ranging from 0 to 1000 μM for 48 h (**A**). U343 and U87 cells were treated with 500 μM TMZ for 48 h (**B**) or for various periods, as indicated (**C**). Total cell lysates were extracted and subjected to immunoblotting with MMP2 and MMP9 antibodies. Original blots are presented in Supplementary Information Fig. 1.

Chemotherapy with TMZ is the current standard for treatment of GBM, but the percentage of recurrent tumors is increasing. In this study, we aimed to study the effects of TMZ on the expression and function of MMPs. First, the cell-killing effect of TMZ was evaluated in U343 and U87MG human GBM cells and GL261 mouse GBM cells using an in vitro MTT cell proliferation assay. All three cells were sensitive to the cytotoxic effect of TMZ up to 50% at drug concentrations of 50–1000 μM (Fig. 1A). To investigate whether TMZ affects MMPs protein expressions, U343 and U87MG cells were treated with final TMZ concentration of 500 μM for 48 h. Western blot results from these cell lysates showed that TMZ increased MMP9 expression but not that of MMP2 (Fig. 1B). Generally, the molecular sizes of MMP9 are ~ 92 kDa in its proform and ~ 82 kDa in its active form^22,23^. While TMZ-induced MMP9 was mainly determined to be ~ 82 kDa, we also observed an overexpressed band close to 75 kDa or multiple bands depending on cell types and MMP9-specific antibodies, as previously reported^24,25^ (data not shown). Therefore, we decided to have the 82-kDa in-gel protein band sequenced (Life Science Laboratory, Seoul, Korea). Protein ID analysis using LC–MS identified that the in-gel protein band contained several fractions of trypsin-digested MMP9 (Supplementary Information Fig. S1). Then, cells were incubated with TMZ for various lengths of time, ranging from 1 to 48 h. The effect of TMZ on MMP9 expression was time-dependent and became apparent after 12 h of TMZ treatment in U343 cells. (Fig. 1C; Fig. S2). These results imply that TMZ, a highly effective anti-cancer drug for GBM upregulates MMP9 expression and thereby may cause unwanted adverse effects in cancer cell invasion and metastasis.

### TMZ activates the transcription of MMP9 in GBM

**Figure 2:**
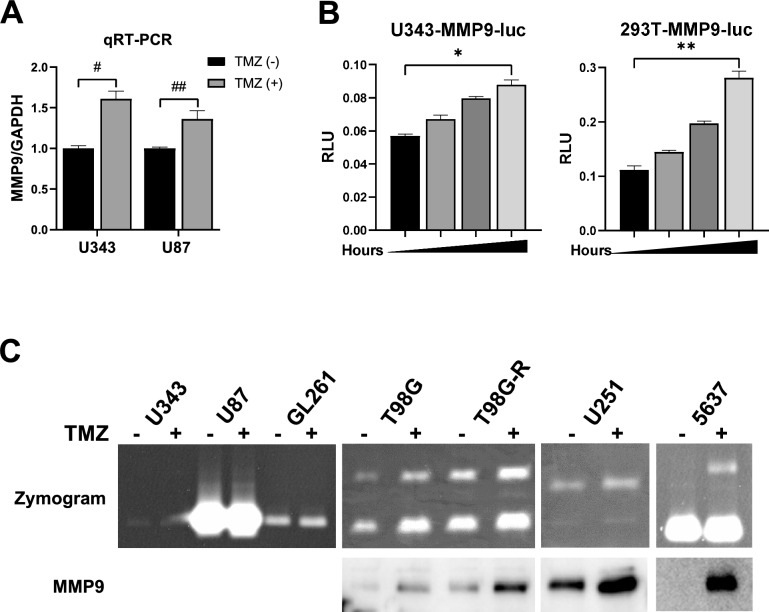
Effects of TMZ on the *MMP9* promoter and its activity in GBM cells. Total RNA of TMZ-treated cells was used for qRT-PCR (**A**). A reporter transcription assay using the *MMP9* promoter region fused to the *Luc* coding region was used to detect *MMP9* transcriptional activity with TMZ treatment from 0 to 48 h in U343 and 293 T cells (**B**). Cells were treated with either 500 μM (U343, U87, and GL261 cells) or 300 μM (T98G, T98G-R, U251, and 5637 cells) of TMZ. Media from the cells were collected, and proteins were separated by non-reducing gelatine SDS-PAGE to demonstrate gelatinase activity in the top panel and subjected to immunoblotting with anti-MMP9 antibodies in the bottom panel (**C**). Each bar represents the mean ± S.D. The difference between two groups was determined by unpaired two-tailed Student's t-test. ^#^*p* = 0.003, ^##^*p* = 0.0254. Dose dependent correaltion was determined by linear regression analysis. *p < 0.001 (r^2^ = 0.8608), **p < 0.001 (r^2^ = 0.9573). Original blots are presented in Supplementary Information Fig. 2.

To examine how TMZ affects *MMP9* transcription, qRT-PCR and reporter assays were conducted. *MMP9* mRNA levels were notably higher in TMZ-treated cells compared with the control cells, especially in the U343 cells (t-test *p* < 0.05; Fig. 2A; Fig. S3). A luciferase reporter plasmid containing the *MMP9* promoter sequence (*MMP9*-*luc*) was transfected into U343 and HEK293T cells, which were used for increasing transfection efficiency, followed by exposure to 500 μM TMZ for various incubation times (Fig. 2B). Luc activity was significantly higher in the TMZ-treated group than the control group and increased linearly in time-dependent manner in both U343 (linear regression analysis r^2^ = 0.8608, *p* < 0.001) and 293 T cells (linear regression analysis r^2^ = 0.9573, *p* < 0.001). To clarify whether TMZ-induced MMP9 expression results in functional activity, an agarose gel-based gelatinase activity was performed after incubating each cell type with 500 μM TMZ for up to 48 h. While MMP9 activity was barely detectable in the media of most GBM cells including U343, U87, and GL261 cells, TMZ upregulated MMP9 activity in T98G and T98G-R (TMZ-resistant T98G cells) and U251 cells. TMZ also upregulated MMP9 activity in 5637 bladder cancer cells (Fig. 2C). The level of MMP2 activity was not changed by TMZ in any of the cells used. Minimal MMP9 activity in most GBM cultured cells seems to be due to the low basal expression level of MMP9 compared to in other cancer cell types as reported at The Human Protein ATLAS portal (www.proteinatlas.org/ENSG00000100985-MMP9)^26^. These results suggest that TMZ-induced MMP9 overexpression is transcriptionally regulated, but that MMP9 activity in vitro function is minimal in GBM cells.

### GBM tumors in mice treated with TMZ overexpress MMP9

**Figure 3:**
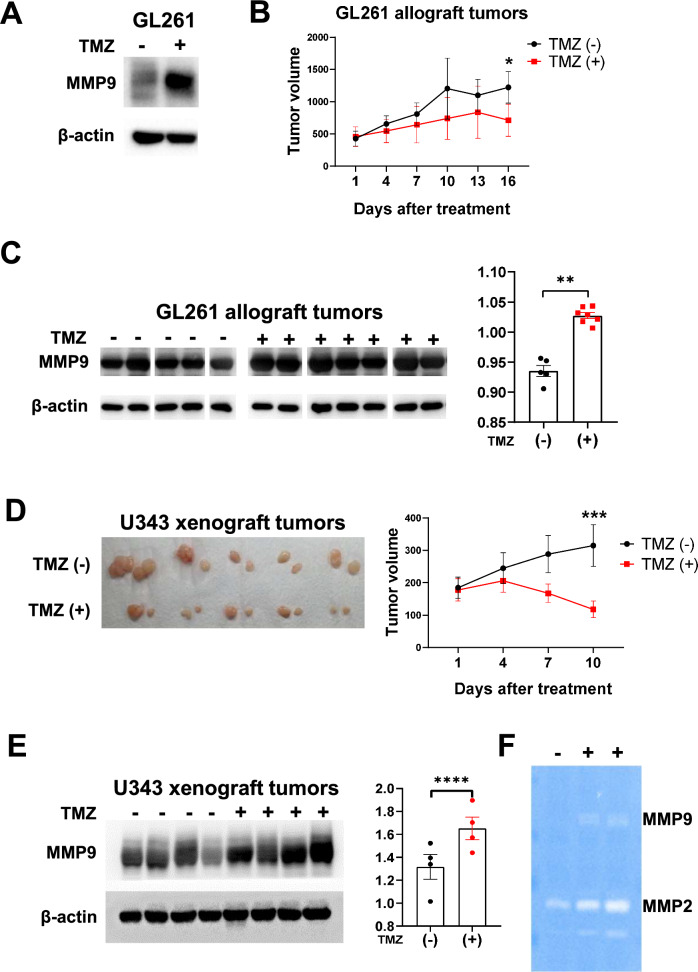
TMZ-induced MMP9 expression in GBM tumor grafts. Effect of TMZ (Sigma-Aldrich) on MMP9 expression in GL261 mouse GBM cells was determined by western blot analysis (**A**). Mice were subcutaneously loaded with GL261 cells followed by inoculation with TMZ. The effect of TMZ on allograft tumor volume is shown (**B**). GL261 allograft tumor tissues were used for MMP9 immunoblotting (left panel of **C**); the results are shown using a bar graph (right panel of **C**). Using TMZ (Shin Poong Pharm), the tumor volume of U343 cell allograft tissue is shown (**D**). Resulting U343 xenograft tumor tissues were evaluated for MMP9 expression (**E**) and MMP-mediated gelatinase activity (**F**). Each bar represents the mean ± S.D. The significance of the difference between cell growth rates was determined by repeated-measures ANOVA. The difference between two groups was determined by paired two-tailed Student's t-test. *p = 0.206, **p < 0.0001, ***p < 0.0001, ****p = 0.0002. Original blots are presented in Supplementary Information Fig. 3.

To investigate whether TMZ induces MMP9 in vivo, GL261 mouse GBM cells were first tested for TMZ-induced MMP9 expression (Fig. 3A). Cells were subcutaneously implanted into C57BL/6 mice, and the mice were treated with either DMSO or 50 mg/kg TMZ (Sigma-Aldrich) for 16 consecutive days. Resulting data showed that tumor volume was generally reduced in the TMZ-treated group (713 ± 659 at day 16) compared with the control group (1223 ± 487 at day 16) without showing significant interaction between groups (repeated-measures ANOVA, *p* = 0.206; Fig. 3B). These ex vivo tumors were then collected and used for western blot analysis, demonstrating that TMZ-treated tumors expressed significantly higher MMP9 protein levels than those in the control group (t-test *p* < 0.0001; Fig. 3C; Fig. S4). We also observed that a few surviving cells from TMZ treatment highly expressed MMP9 by immunohistochemical staining (Fig. S5). To reproduce TMZ-induced MMP9 stimulation in vivo using U343 human GBM cells and a clinically used formulation of TMZ (Shin Poong Pharm, Seoul, Korea), a small-scale animal study was conducted by implanting cells into athymic nude mice. There was a very significant difference in tumor volume between groups and days (repeated-measures ANOVA, *p* < 0.0001; Fig. 3D). In these ex vivo tumors, TMZ also caused MMP9 overexpression (t-test *p* = 0.0002; Fig. 3E). We expected MMP9 expression in tumors were quite low and overloaded proteins for Western blot analysis. Since total proteins from ex vivo tumors were very limited especially in TMZ-treated group, we had limitations to repeat same experiments. While several MMP9 bands from graft tumors were saturated (Fig. 3C and E), most were found to be in TMZ-treated tumors suggesting that signal saturation did not affect outcome of results and ex vivo tumors from TMZ-treated mice promoted expression of MMP9. Selected tumor tissues were also analyzed for MMP’s gelatinolytic function using a zymography assay (Fig. 3F). Both MMP9 and MMP2 was increased in TMZ-treated tumors. In cultured cell conditions, TMZ only upregulated expression of MMP9, not MMP2 as shown in Fig. 1B. We assume that the microenvironment in tissue is different from that of the cell culture environment. For example, MMPs are also highly expressed in vascular endothelial cells, blood cells, and stromal cells, which are usually highly populated in cancer tissues. Regardless, these results suggest that the anti-cancer agent TMZ promotes the expression and activity of MMP9 in vivo, and that prolonged TMZ treatment can cause adverse effects in GBM patients by stimulating metastasis through activation of MMP9.

### TMZ regulation of the MMP9 promoter is mediated through MAPK pathways

**Figure 4:**
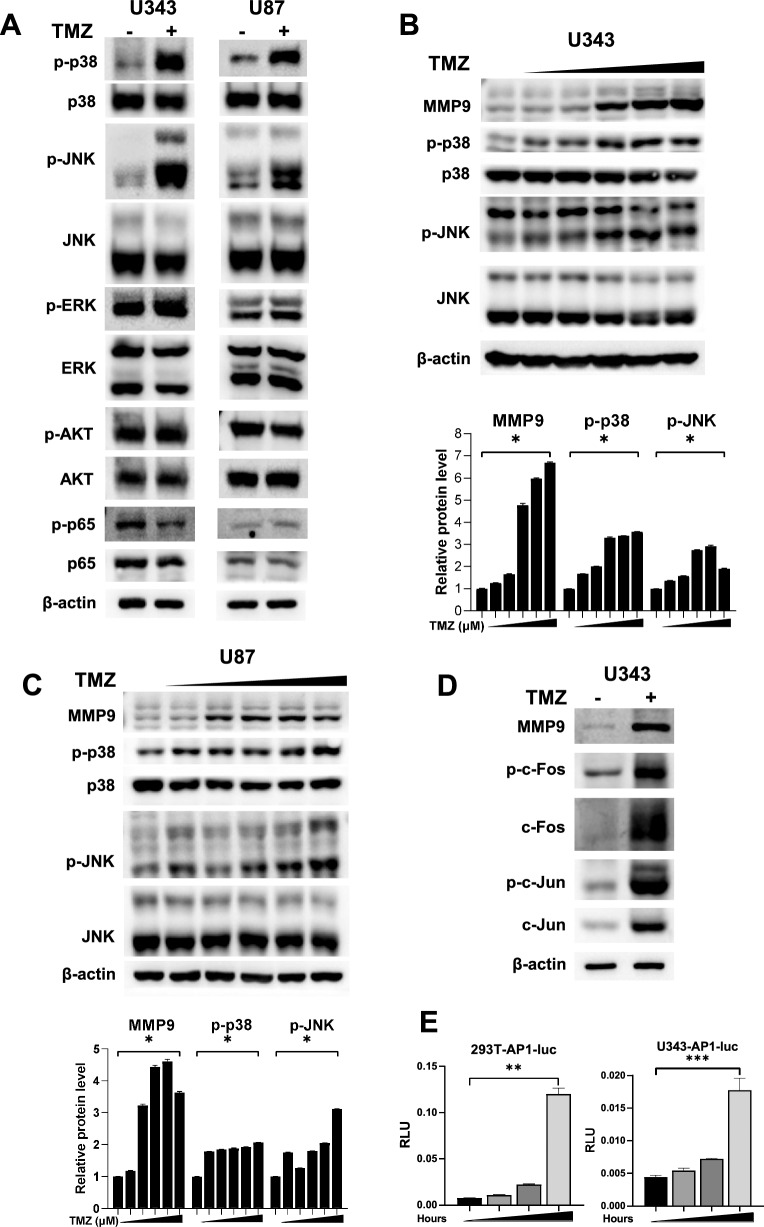
Effects of TMZ in the regulation of selected signaling pathways. Both U343 and U87 cells were treated with TMZ (500 μM) for 48 h followed by western blot analyses for proteins, as indicated (**A**). Either U343 (**B**) or U87 (**C**) cells were also treated with various doses (0 to 500 μM) of TMZ followed by western blot analysis. The bottom panels of B and C show quantitated proteins. Phospho-forms of p38 and JNK were normalized to the corresponding total proteins. The effect of TMZ on c-Fos and c-Jun expression was shown in U343 cells (**D**). The effect of TMZ on AP-1 activity was investigated by a reporter transcription assay in both 293 T and U343 cells, as described in Fig. 2B (**E**). Each bar represents the mean ± S.D. The difference between two groups was determined by paired two-tailed Student's t-test. *p < 0.0001, **p = 0.0004, ***p = 0.005. Original blots are presented in Supplementary Information Fig. 4.

To elucidate how TMZ regulates *MMP9* transcription, we investigated the roles of several major signalling pathways, including the MAPK and NF-κB pathways, in U343 and U87 cells. It has been widely understood that MAPK and PI3K/NF-kB pathways are major regulators of MMP9 regulation^7,9,27,28^. In both U343 and U87 cells, TMZ treatment induced hyperphosphorylation of p38 and JNK, but not ERK or AKT (Fig. 4A). TMZ had little effect on the transcription factor p65, a subunit of the Nuclear factor kappa B (NF-κB) complex in cells that survived TMZ toxicity. Furthermore, both cell types treated with TMZ ranging from 0 to 500 μM showed gradual increases in MMP9 protein expression accompanied by increased phosphorylation of p38 and JNK (t-test *p* < 0.0001; Fig. 4B–C), suggesting that TMZ increases MMP9 expression through one or more cascades of the MAPK signaling pathways. We further analyzed downstream signaling proteins of MAPKs that play important roles in the transcription of target genes. The AP-1 transcription factor is actively involved in promoter regions of MMP9 and is also major target of MAPKs^8,29^. AP-1 consists of c-Fos and c-Jun heterodimers. TMZ also upregulated the expression of total c-Fos and c-Jun and their subsequent phosphorylation (Fig. 4D). Phosphorylation of p38 stimulates total c-Jun and c-Fos expression as well as their phosphorylation, and AP-1 is responsible for p38-controlled regulation of MMP9 expression^8^. In addition, a reporter transcription assay using *AP-1*-*luc* demonstrated that TMZ stimulated AP-1 activity in both 293 T and U343 cells (t-test *p* = 0.0004 and *p* = 0.0049, respectively; Fig. 4E). The results demonstrate that TMZ activates the phosphorylation of MAPKs, including p38 and JNK, and subsequently activates c-Fos and c-Jun phosphorylation to further stimulate MMP9 transactivation.

### TMZ induces MMP9 expression through the p38 and JNK MAPK pathways

**Figure 5:**
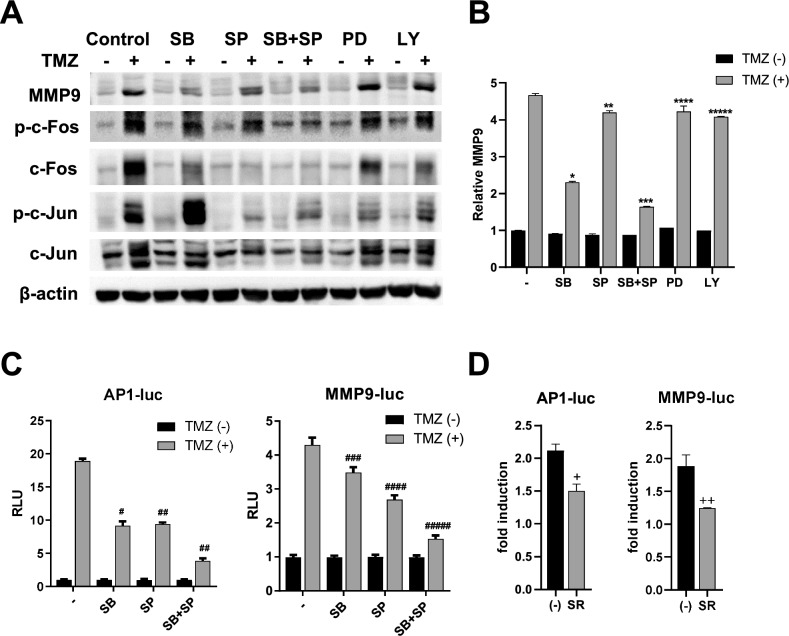
Effects of inhibitors on TMZ activation of p38 and JNK. U343 cells were pretreated with the various inhibitors indicated, followed by 500 μM TMZ treatment for 48 h and analyzed by western blot analysis (**A**). Densitometric representation of MMP9 expression is shown as a bar graph (**B**). The reporter gene transcription assay demonstrates the effect of the SB and SP compound (**C**) or AP-1 specific inhibitor (SR11302 compound) (**D**) on the TMZ-mediated transcriptional activities of MMP9 and AP-1. Values indicate either relative luciferase unit (RLU) (luc/renilla) or fold induction (RLU/basal activity without TMZ). Each bar represents the mean ± S.D. The difference between two groups was determined by paired two-tailed Student’s t-test. *p < 0.0001, **p = 0.002, ***p < 0.0001, ****p = 0.083, *****p = 0.0002, ^#^*p* = 0.0002, ^##^*p* < 0.0001, ^###^*p* = 0.038, ^####^*p* = 0.0029, ^#####^*p* = 0.0003, ^+^*p* = 0.0145, ^++^*p* = 0.0215. Original blots are presented in Supplementary Information Fig. 5.

MAPK pathways are activated by a variety of extracellular and intracellular factors, including genotoxic stress. In glioblastoma cells, DNA alkylation induced by TMZ stimulates MAPK signaling pathways and leads to a signaling cascade that transmits information to the cell nucleus, ultimately triggering target gene expression^7,9^. To determine whether hyperphosphorylation of p38 and JNK by TMZ is linked to the stimulation of *MMP9* expression, inhibitors of p38 (SB), JNK (SP), ERK (PD), or PI3K (LY) were incubated with U343 cells pretreated with TMZ. Western blot analysis demonstrated the effects of each inhibitor on TMZ-mediated expression of MMP9 and c-Fos and c-Jun (Fig. 5A). The data from Fig. 5A were quantitated, and the results are shown in Fig. 5B. Western blot anlysis demonstrated that elevated expression of MMP9 induced by TMZ was significantly reduced in the presence of SB202190, a p38-specific inhibitor (t-test *p* < 0.0001), and slightly reduced by SP600125, a JNK-specific inhibitor (t-test *p* = 0.0019). Simultaneous application of the SB and SP compounds further reduced TMZ-induced *MMP9* expression (t-test *p* < 0.0001). However, specific inhibitors targeting ERK (PD) and PI3K (LY) minimally affected TMZ-induced MMP9 levels (t-test *p* = 0.083 and *p* = 0.0002, respectively). At the same time, TMZ-induced hyperphosphorylation of c-Fos and c-Jun diminished with the application of SB, SP, or SB + SP (Fig. 5A). Then, a reporter-mediated transcription assay was performed to test whether the SB, SP, or both compounds counteract TMZ-mediated AP-1 transactivation. In HEK293T cells, both SB and SP compounds significantly inhibited AP-1 activity (t-test *p* = 0.0002 and *p* < 0.0001, respectively) (Fig. 5C, left panel). SB and SP also downregulated MMP9 promoter acitivity induced by TMZ (*p* = 0.038, *p* = 0.0029, respectively) (Fig. 5C, right panel). Treatment of both compounds further reduced promoter activities of AP-1 and MMP9 (t-test *p* < 0.0001 and *p* = 0.0003, respectively). SR11302, an AP-1 inhibition-specific retinoid, significantly inhibits both 12-O-tetradecanoylphorbol-13-acetate-induced papilloma formation and AP-1 activation in 7,12-dimethyl benz(a)anthracene-initiated mouse skin^30^. In this study, SR11302 significantly inhibited promoter activities of both AP-1 and MMP9 promoted by TMZ (t-test *p* = 0.0145 and *p* = 0.0215, respectively) (Fig. 5D). These results clearly demonstrate that TMZ activates the p38 and JNK pathways, which affect the transcription factor AP-1, thereby inducing MMP9 overexpression in glioblastoma. Together, these data suggest that GBM cells that escape death by TMZ toxicity highly express MMP9 as an adverse effect. MMP9 upregulation occurs via the p38 MAPK and JNK signalling pathways that targets the AP-1 transcription factor; many studies have shown that this pathway contributes to cancer development and progression.

## Discussion

TMZ is widely used as a first-line drug for GBM as well as recurrent anaplastic astrocytoma and metastatic melanoma^31,32^. TMZ operates by introducing DNA damage in cancer cells, primarily through the methylation of DNA at the O^6^ position of guanine. This leads to DNA strand breaks and ultimately cell death. However, at least 50% of TMZ-treated patients do not respond to TMZ^1^. TMZ resistance is not mediated via a single molecular event but by multiple pathways, some of which remain unclear. Possible causes of GBM recurrence are the strongly invasive nature of GBM cells; chemo- and radio-resistance; the high degree of vascularization, heterogeneity, and reduction of chemotherapeutic drug effusion due to the blood–brain barrier; and heterogeneity of the tumor microenvironment^33^. In this report, we demonstrated that GBM cells, including GL261, U87, and U343 cells, were effectively killed by 500 µM TMZ, as demonstrated by previous reports^34,35^. GBM cells that survived TMZ-mediated cytotoxicity promoted MMP9 expression and its activity both in vitro and ex vivo. MMP2 and MMP9 play important roles in GBM invasion and are associated with glioma malignancy^15–19^. MMP9 expression is also correlated with glioma grade and correlated with survival outcomes^20,36,37^. Hence, MMP9 is an independent prognostic factor for primary GBM, and GBM patients with low MMP9 expression benefit from TMZ chemotherapy^20^. In this report, we demonstrated that short-term exposure of TMZ in GBM cells surprisingly upregulates expression of MMP9. Unlike our observation, altered expression of MMP9 by TMZ in GBM has not been observed by several^21,38^. Cancer therapeutic agents including TMZ have been reported to induce senescence. Alkylating agents can alkylate DNA that leads to DNA damage during DNA replication. Cisplatin and TMZ are common alkylating agents that can promote cellular senescence^39^. These senescent cells contribute to tumor development through various pathways including NF-κB, C/EBPβ, and p38MAPK pathways. Cellular senescence is reported to promote MMP9 expression^40,41^. The highly invasive senescent cells are shown to have activated STAT3 caused by increased secretion of IL-6 and IL-8, resulting in the upregulation of MMPs including MMP9^39^. At the same time, several studies also demonstrated that the senescent cells did not significantly promote expression of MMP9^42,43^. TMZ-induced senescence is highly inconsistent according to glioma cell lines and strongly dependent on the expression of MGMT^44–48^. The accumulation of SA-β-gal activity, a biomarker of cellular senescence, rises after TMZ treatment for 6 days: about 50% senescent cells in LN229 and 0–15% in U87, U138, GaMG, and LN308 cells^44–47^. It has been also reported that TMZ did not mediate the senescence in some GBM cells at elevated TMZ concentrations^48^. In our study, the elevated MMP9 by TMZ was observed from 12 h after exposing to TMZ and obviously overexpressed in 24–48 h. MMP9 induced by TMZ were observed in cells regardless of MGMT status. Therefore, further studies need to be done to clarify that our observation on TMZ-mediated promotion of MMP9 expression is mediated through cellular senescence induced by anti-cancer agent.

Several intracellular pathways are involved in the growth and survival of GBM cells; these include activation of the epidermal growth factor receptor (EGFR)^49^. The EGFR is one of four members of the Erb family of tyrosine kinase receptors, which mediate intrinsic intracellular protein tyrosine kinase activity^50^. The activation of EGFR leads to downstream signaling pathways, such as the phosphorylation of AKT, MAPK, and JNK^7^. The promoter region of *MMP9* possesses multiple transcription factor binding motifs, including ones for NF-κB, SP-1, Ets, AP-1, and retinoblastoma (RB)^51^. TMZ-induced DNA damage activates AP-1, composed of c-Jun and c-Fos, NF-κB, and early growth response (egr)-1 protein^52–55^. These transcription factors can bind to the *MMP9* promoter and induce gene transcription and its activity^51,56^. However, the relationship between TMZ treatment (or resistance) to GBM and MMP9 transcription and activity has been unclear. In this report, we demonstrated that TMZ activated some EGFR kinase activities, namely those of p38MAPK and JNK, in GBM cells. TMZ stimulated hyper-phosphorylation of both p38 and JNK but not of ERK and AKT. NF-κB was not activated by TMZ either. Treatment of GBM cells with TMZ also stimulated the expression of c-Fos and c-Jun and their subsequent phosphorylation. Specific inhibitors of p38 and JNK downregulated TMZ-induced MMP9 activity through inhibition of c-Fos and c-Jun expression, suggesting that TMZ activates MMP9 by specifically stimulating p38 MAPK and JNK kinase activity.

Several studies have proposed to link TMZ and MMP9 by a way of MAPK signaling^21,49,53^. While experiments in these studies were limited to RT-PCR and gelatin zymography to show TMZ-mediated MMP9 regulation, TMZ was shown to no effect on *MMP9* transcription and its activity in GBM cells^21^. Source of TMZ should be considered to be a variable factor for producing reliable data since most studies have been done using commercially available TMZ and in our case TMZ from Sigma-Aldrich failed to yield consistent data in terms of the induction of MMP9 expression (Fig. S6). Therefore, we used clinically tested TMZ (Shin Poong Pharm) for our entire study demonstrating that TMZ upregulates MMP9 expression both in vitro and ex vivo models. In most GBM cells, MMP9 expression and its activity were not detectable or very limited as shown in Fig. 2C. Enhancement of MMP9 activity by TMZ was restricted to only several GBM cells including T98G and U251. TMZ also activated MMP9 expression in T98G and U251. While MMP9 expression is upregulated in cancer tissues and pregressed GBM, MMP9 expression is mainteained at low level in most GBM cells as presented at The Human Protein ATLAS portal (www.proteinatlas.org/ENSG00000100985-MMP9)^26^. Despite identical histopathology, as a matter of fact, GBMs are not a homogeneous group of tumors. Compared to MMP2, activity of MMP9 is weak in many GBM cells and in patients^16,57^. In GBM cell lines, MMP2 was highly expressed in most cells; however, MMP9 expression remained controversial among various studies as reviewed by Hagemann et al^16^. Extensive quantitative RT-PCR also demonstrated the presence of MMP2 and negligibly small amounts of MMP9 mRNA in GBM cells including U251, U87, T98, A172, and U138^58^. Subsequent zymography assays of the baseline expression demonstrated that latent and active forms of MMP2 were present in both U87 and T98, and there was no observable MMP9 activity. TMZ-induced MMP9 activity in our study may not be enough for gelatinase activity compared to some cells (eg, melanomas, squamous cell carcinomas) possesing high MMP9 basal level. Follow-up in vivo studies using syngenic GL261 cells and human U343 cells supported that cancer cells that escaped TMZ-mediated cytotoxicity promoted the expression of MMP9. In these TMZ-treated graft tumors, MMP9 was significantly upregulated with promoted MMP9 activity. These results suggest that TMZ-resistant cells somehow acquire their invasive phenotypes through MMP9 activation.

**Figure 6:**
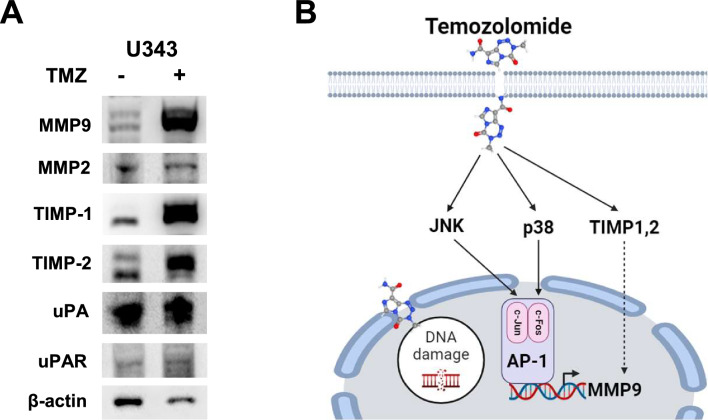
Model of TMZ-induced *MMP9* upregulation in GBM cells. (**A**) U343 cells were analyzed for TMZ-medated regulation of several protease inhibitors by western blot analysis. (**B**) Here we propose the following hypothesis: when GBM cells are exposed to TMZ, some cells survive TMZ-mediated cell cytotoxicity. These cells acquire invasive potential after surviving TMZ cytotoxicity. TMZ stimulates phosphorylation of both p38 MAPK and JNK and activates the expression and phosphorylation of c-Jun and c-Fos to further transactivate AP-1 sites in the *MMP9* promoter region. Since TMZ also affects the expression of TIMP proteins, associations between MMP9 and other proteases may determine the degree of invasiveness of TMZ-treated cancer cells. This model was created with BioRender.com. Original blots are presented in Supplementary Information Fig. 6.

In our study, TMZ clearly activated many EGFR downstream signaling pathways, including those of p38, JNK, and AP-1, shown by others^53,55,56^, however, basal MMP9 expression levels were below the detection limit in most GBM cells, as shown in Fig. 2C. Nature of low basal MMP9 level in GBM cells can result in extremely difficult technical issues. It can be more challenging that cells showed variable size of cleaved MMP9, being 65 kDa, 72 kDa, or 82 kDa depending on cell types and culture conditions, as previously described^23–25^. During our searching process for TMZ-mediated protease regulation, we have also observed that TMZ induced the expressions of tissue inhibitor of metalloproteinase TIMP-1 and TIMP-2 in U343 cells (Fig. 6A). MMP2, uPA, and uPAR remained unchanged under TMZ treatment conditions. TIMP-1 is an inhibitory molecule that regulates MMPs by interacting with their N-termini to form stable complexes, so that the pro-enzymes cannot cleave the pro-domains to release the active forms^59^. TIMPs have various biological activities, such as in the modulation of cell proliferation, cell migration and invasion, anti-angiogenesis, pro- and anti-apoptosis, and synaptic plasticity, many of which are independent of metalloproteinase inhibition^59^. Increased *TIMP-1* expression indicates poor prognoses in brain, lung, breast, prostate, colon, and several other cancers^60^. Whereas MMP9 overexpression associated with glioma malignancy is accompanied by TIMP-1 overexpression^61^, the expression status of TIMP-1 and TIMP-2 determined during glioma progression have been highly controversial, suggesting that balance between the expression and/or activity of MMPs and their specific inhibitory proteins may decide glioma progression, including the processes of migration and invasion. This may explain why our attempts were failed to verify whether TMZ-induced MMP9 upregulation functions to promote GBM cell migration and invasion using transwell membranes (Fig. S7). Therefore, it is plausible that TMZ regulates several proteins involved in regulating metalloproteinase expression and activity; association of MMP9 with such proteins may have affected MMP9-mediated gelatin digestion in our study, resulting in marginal MMP9 activity compared with strong TMZ-mediated MMP9 expression.

In summary, TMZ primarily functions as a DNA-damaging chemotherapeutic agent used to treat GBM. GBM expresses greater amounts of MMPs compared with low-grade gliomas. Our data suggest that GBM cells that escape death by TMZ toxicity more highly express MMP9, and possibly TIMP-1 and TIMP-2, as poor prognostic factors than cells not exposed to chemotherapy. This report does not suggest that there are tight connections between MMP9 and TMZ resistance in GBM, which needs to be clarified in subsequent studies. We simply show that transient exposure of TMZ to GBM cells promotes expression of MMP9 and its response is varied in different cells regardless of status of TMZ response or MGMT expression status. MMP9 upregulation occurs via targeting of AP-1 transcription factors by the p38 MAPK and JNK signalling pathways, which many studies have shown contribute to cancer development and progression (Fig. 6B). While TMZ may not directly target MMP9 or the EGFR pathway, chemotherapy-induced stress and DNA damage can activate various cellular signaling pathways, in this case the p38 MAPK and JNK signalling pathways, as part of the cellular stress response. This study demonstrates that GBM cells responding to TMZ exert adverse effects by promoting MMP9 activity, and we also propose a mechanism for TMZ-mediated MMP9 regulation, which certainly leads to promoted cancer metastasis from TMZ resistant recurrent GBM.

## Materials and methods

### Cell lines and cell culture

Human glioblastoma cell lines U343 (RRID:CVCL_S471), U87 (RRID:CVCL_0022), U251 (RRID:CVCL_0021), T98G (RRID:CVCL_0556), the mouse glioblastoma cell line GL261 (RRID:CVCL_Y003) and human bladder cancer cell line 5637 (RRID:CVCL_0126) were purchased from the American Type Culture Collection (ATCC, Manassas, VA, USA). Cells were cultured in Dulbecco’s modified Eagle’s medium (DMEM; Welgene, Korea). The complete medium was supplemented with 5% fetal bovine serum (FBS, Gibco Life Technologies, Grand Island, NY, USA) and 1% penicillin/streptomycin (Gibco Life Technologies) before use. All cultures were maintained at 37 °C in 95% air and 5% CO_2_ in a culture hood, and the medium was renewed every 3 days. The 5637 bladder cancer cells and HEK293T cells (RRID:CVCL_0063) have been described previously^62^.

### Reagents

TMZ was used in this study from two different sources. TMZ in the name of Temodar from Shin Poong Pharm (Seoul, Korea) was provided by Chonnam National University Hwasun Hospital. Another TMZ was purchased from Sigma-Aldrich (St. Louis, MO, USA) TMZ from Shin Poong Pharm was used in most of the experiments performed in this study, except for the animal study using GL261 cells. Antibodies used in this study are provided in Supplementary information Table S1. A selective p38 MAP kinase inhibitor SB202190 is purchased from Sigma-Aldrich. A JNK inhibitor SP600125 is from Selleck Chemicals (Houston, Texas, USA). An ERK/MEK inhibitor PD98059 is from MedChemexpress MCE (Princeton, NJ, USA). A PI3K/AKT inhibitor LY294002 is from BioVision Inc (Milpitas, CA, USA). An AP-1 inhibitor SR11302 is from Tocris Bioscience (Minneapolis, MN, USA).

### Cell viability assays

The growth inhibitory effect of TMZ in vitro was analyzed by the MTT in vitro proliferation assay. Five thousand cells were seeded in each well of a 24-well plate. The next day, cells were treated with various concentrations of TMZ (from 0 to 1000 µM) and cultured for up to 3 days. TMZ (Shin Poong Pharm). Cells were exposed to MTT solution and incubated for 4 h. The crystal obtained was dissolved by DMSO, and the absorbance was measured at 570 nm using an Epoch microplate spectrophotometer with Biotek Gen5 software (Agilent Technologies, Santa Clara, CA, USA).

### Animal studies

Six-week-old male athymic nude mice (Orient Bio Co., Ltd., Korea) were housed under specific pathogen-free conditions in the animal facility at the Hwasun Biomedical Convergence Center. All procedures performed in studies were approved by the Animal Use and Care Committees at Chonnam National University Medical School and in accordance with our institution’s guidelines for animal care and with the 1964 Helsinki declaration and its later amendments or comparable ethical standards. U343 cells were injected subcutaneously into the flanks of nude athymic mice (2 × 10^7^ cells for each tumor). When the tumor volumes reached approximately 180 mm^3^, mice were randomly allocated to one of two groups (five mice per group). Mice from each group were administered DMSO or 50 mg/kg TMZ (Shin Poong Pharm) intraperitoneally for 10 consecutive days. In addition, GL261 mouse cells were injected subcutaneously into the flanks of C57BL/6 mice (10^7^ cells for each tumor). When the tumor volumes reached approximately 180 mm^3^, mice were randomly allocated to one of two groups and administered DMSO or 50 mg/kg TMZ (Sigma-Aldrich) intraperitoneally for 15 consecutive days. Every 3 days, tumor size was measured and calculated using the following equation: (length × width^2^)/2.

### Western blotting

Total protein lysates (15 μg) were separated on a 7.5–10% SDS–polyacrylamide gel and then transferred to Immobilon-P membrane (Millipore, Billerica, MA, USA). The membranes were blocked with 3% bovine serum albumin and then cut depending on protein sizes before hybridization with primary antibodies at 4 °C overnight. The bands were visualized and analyzed using the Immobilon Western detection system (Millipore, Billerica, MA, USA) and ChemiDOC MP Gel Imaging System (Bio-Rad, Hercules, CA, USA).

### Zymography assay

Serum-free conditioned media from cancer cells cultured in the presence or absence of TMZ were prepared in non-reducing conditions and separated on a 7.5% SDS–polyacrylamide gel containing 1% gelatin (G9382, Sigma-Aldrich). The gel was incubated in renaturing buffer to remove SDS following by rinsing with dH_2_O, and enzymatic activity to visualize the bands was developed overnight. The gel was stained with Coomassie blue R-250 staining solution. The gelatinolytic bands appeared as clear bands on a dark background after appropriate destaining.

### Luciferase promoter assay

Cells were transfected with a luciferase reporter plasmid containing the promoter sequence of *MMP9* or *AP-1* using Lipofectamine 2000 (Invitrogen, Waltham, MA, USA) with 0.1 μg of reporter and 2 ng of renilla luciferase reporter as described in the manufacturer's protocol. The MMP9-luc promoter contains promoter region of MMP9 (-924/ + 13) in pGL4.17[luc 2/Neo] vector. AP1-luc was purchased from Stratagene (Santa Clara, CA, USA). The transfected cells were grown in media with or without TMZ for up to 48 h. When necessary, the transfected cells were pretreated with specific inhibitors, as indicated, for 3 h and then grown in media with or without TMZ for the designated amounts of time. The light signals of luciferin were detected using the Dual-Luciferase Reporter Assay System (Promega, Madison, WI, USA) and a Glomax Navigator instrument (Promega).

### RT-PCR

Total mRNA from U343 and U87 cells was isolated using TRIzol (Life Technologies) and quantified by a NanoDrop spectrophotometer. Then 1 μg of extracted RNA and 50 pM Oligo dT were preheated at 72 °C for 10 min. Reverse transcription was conducted with a M-MLV Reverse Transcriptase kit (Promega) at 42 °C for 1 h to obtain cDNA. Total cDNA was used for qRT-PCR followed TOPreal™ SYBR Green qPCR PreMIX protocol (Enzynomics co Ltd, Daejeon, Republic of Korea). PCR was performed with 1 μl template cDNA using PCRBIO Taq DNA Polymerase & Mixes kit (PCR Biosystems Inc., PA, USA). The forward and reverse primers were designed using Vector NTI software. Primers used included human MMP9 (Fw 5'-CATCGTCATCCAGTTTGG-3'; Rv 5'-GATGGATTGGCCTTGGAA-3'), human GAPDH (Fw 5'-GAAGGTGAAGGTCGGAGTC-3'; Rv 5'-GAAGATGGTGATGGGATTTC-3'). Amplification reactions were performed using a DNA thermal cycler (Eppendorf, Hamburg, Germany). The 10 μl of PCR products were separated by 1.5% agarose gel electrophoresis.

### Statistics

The results were analyzed using either Prism 5 (La Jolla, CA, USA) or SPSS 21 (IBM, Chicago, IL, USA). Prism was used to produce graphs. Student's t-test was used to compare the difference between 2 groups. The difference in growth rate between the two groups were determined by repeated measures analysis of variance (ANOVA). Differences were considered significant when p value calculated less than 0.05.

### Ethical approval and consent to participate

All procedures performed in studies were approved by the Animal Use and Care Committees at Chonnam National University Medical School and in accordance with our institution’s guidelines for animal care and complied with ARRIVE guidelines.

### Supplementary Information

Supplementary Information 1.

Supplementary Information 2.

## Data Availability

The data generated in this study are available within the paper and Supplementary information. The datasets used and/or analyzed during the current study are available from the corresponding author upon reasonable request.
