# Supplementary material for: Temozolomide promotes matrix metalloproteinase 9 expression through p38 MAPK and JNK pathways in glioblastoma cells
Source: Sci Rep. 2024 Jun 21;14:14341. doi: 10.1038/s41598-024-65398-2 (PMC11192740; doi:10.1038/s41598-024-65398-2)
Supplement: Supplementary file 1 — Supplementary Information 1. [file 41598_2024_65398_MOESM1_ESM.pdf]

Temozolomide promotes matrix metalloproteinase 9 expression through p38 MAPK and JNK pathways in glioblastoma cells

Hien Duong Thanh[1], Sueun Lee[1,2], Thuy Thi Nguyen[1], Thang Nguyen Huu[3], Eun-Jung Ahn[4], Sang-Hee Cho[5], Min Soo Kim[6], Kyung-Sub Moon[4], Chaeyong Jung[1*]

Figure S1

< Sequence matching >

1. JCY_82kDa (1st ID)

N	Unused	Total	% Cov	Accession #	Name	Species	Peptides(95%)
1	8	8	35.1	EAW75776.1			4

< Sequence coverage >

- **Prot N1 : minor protein ID** https://www.ncbi.nlm.nih.gov/protein/EAW75776.1
- MSLWQPLVLVLLVLGCCFAAPRQRQSTLVLFPGDLRTNLTDRQLAEEYLYRYGYTRVAEMRGESKSLGPALLLL
  QKQLSLPETGELDSATLKAMRTPRCGVPDLGRFQTFEGDLKWHHHNITYWIQNYSEDLPRAVIDDAFARAFALW
  SAVTPLTFTRVYSRDADIVIQFGVAEHGDGYPFDGKDGLLAHAFPPGPGIQGDAHFDDDELWSLGKGVVVPTRF
  GNADGAACHFPFIFEGRSYSACTTDGRSDGLPWCSTTANYDTDDRFGFCPSERLYTRDGNADGKPCQFPFIFQG
  QSYSACTTDGRSDGYRWCATTANYDRDKLFGFCPTRADSTVMGGN**SAGELCVFPF**TFLGKEYSTCTSEGRGDGR
  LWCATTSNFDSDKKWGFCPDQGYSLFLVAAHEFGHALGLDHSSVPEALMYPMYRFTEGPPLHKDDVNGIRHLYG
  PRPEPEPRPPTTTTPQPTAPPTVCPTGPPTVHPSERPTAGPTGPPSAGPTGPPTAGPSTATTVPLSPVDDACNV
  NIFDAIAEIGNQLYLFKDGKYWRFSEGR**GSRPQGPFLIADK**WPALPRKLDSVFEEPLSKKLFFFSGRQVWVYTG
  ASVLGPRRLDKLGLGADVAQVTGALR**SGRGKMLLFSGRRLWRFDVK**AQMVDPRSASEVDRMFPGVPLDTHDVFQ
  YREKAYFCQDRFYWRVSSRSELNQVDQVGYVTYDILQCPED
- **Confidence : Green > Yellow > Red, Gray : not detected.**

Figure S1. Protein ID analysis using LC-MS identified that the in-gel protein band contained several fractions of trypsin-digested MMP9

Figure S2

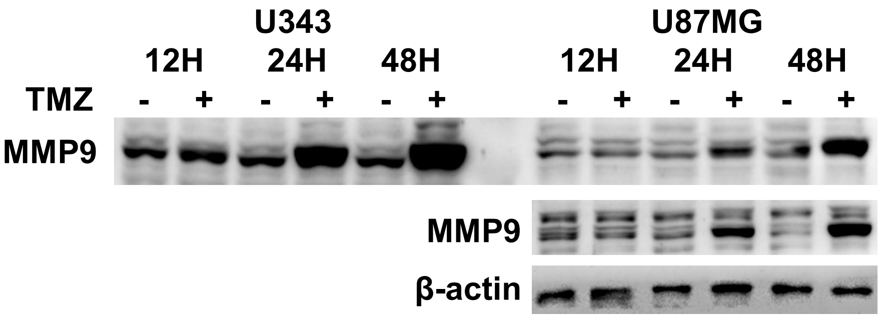

Figure S2. Time-dependent upregulation of MMP9.
U343 and U87 cells were treated with 500 μM TMZ for various periods, as indicated. Original blots are presented in Supplementary Information Figure S2.

Figure S3

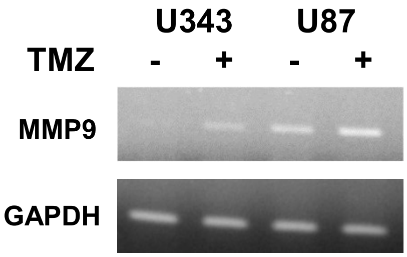

Figure S3. Effects of TMZ on the *MMP9* transcription. Total RNA of TMZ-treated cells was used for RT-PCR followed M-MLV Reverse Transcriptase kit (Promega) and PCRBIO Taq DNA Polymerase & Mixes kit (PCR Biosystems) protocols.

Figure S4

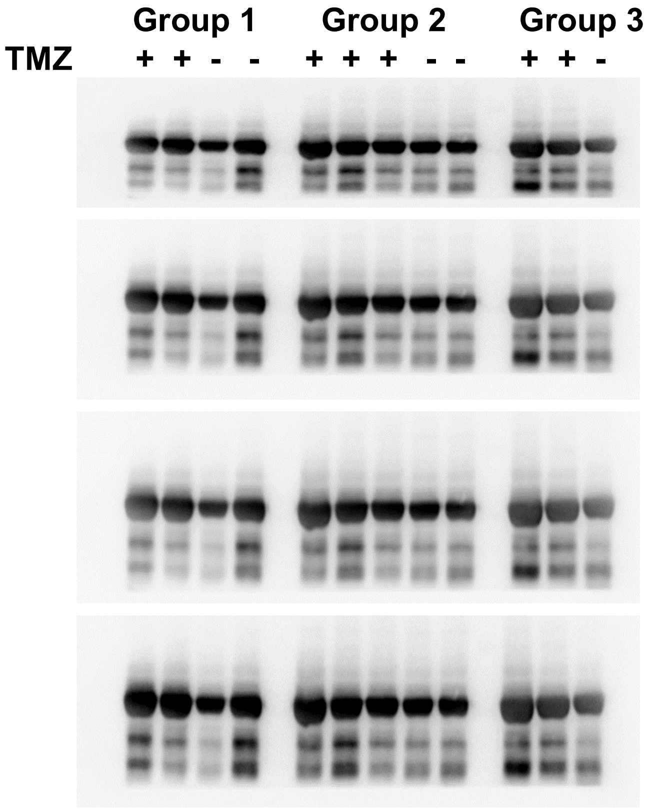

Figure S4. TMZ effects on MMP9 expression in GL261 tumor. As shown in Fig. 3C, GL261 allograft tumor tissues were used for MMP9 immunoblotting and exposed at different time points. Original blots are presented in Supplementary Information Figure S4.

Figure S5

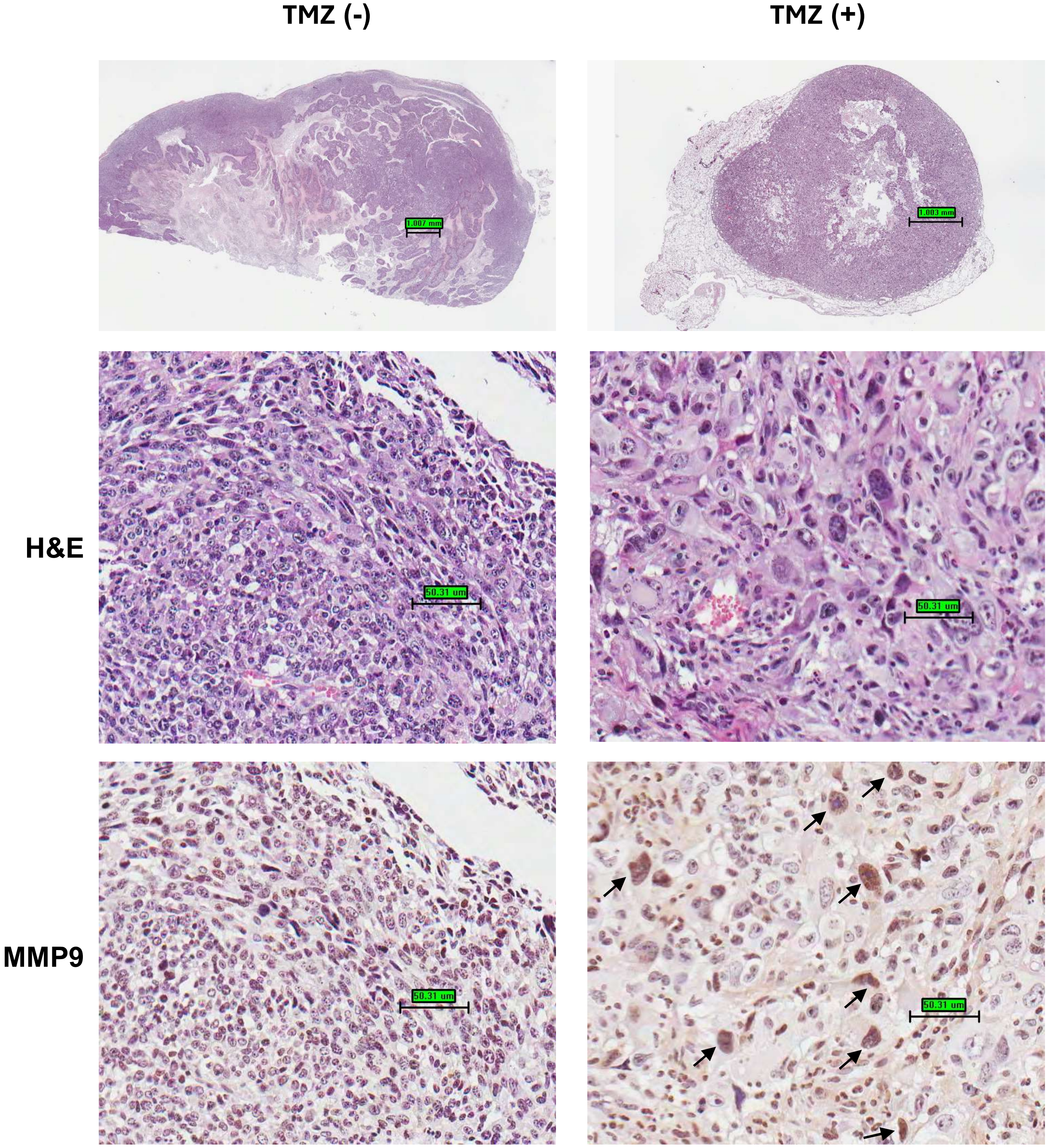

Figure S5. TMZ effects on MMP9 expression in GL261 tumor. GL261 allograft tumor tissues were shown by hematoxylin and eosin staining and analyzed for MMP9 localization by immunohistochemistry using MMP9 antibodies from Sigma-Aldrich (#AV33090, St. Louis, MO, USA). Some of TMZ-treated surviving cells are highly positive for MMP9 expression (black arrows). Some are nuclear and perinuclear. On the other hand, TMZ-untreated tumor cells showed no or minimal staining of MMP9. The scale bar of images in the first row is 1 mm, and in the remaining images is 50 μm.

Figure S6

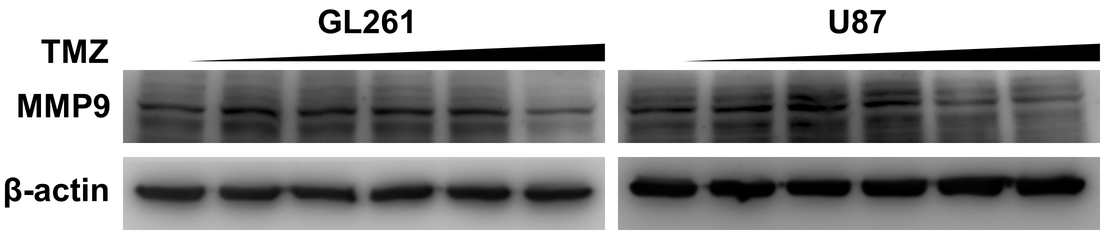

Figure S6. Induction of MMP9 expression by TMZ (Sigma-Aldrich) in GL261 and U87 cells at the range of concentration from 0 – 300 μM. Original blots are presented in Supplementary Information Figure S6.

Figure S7

A

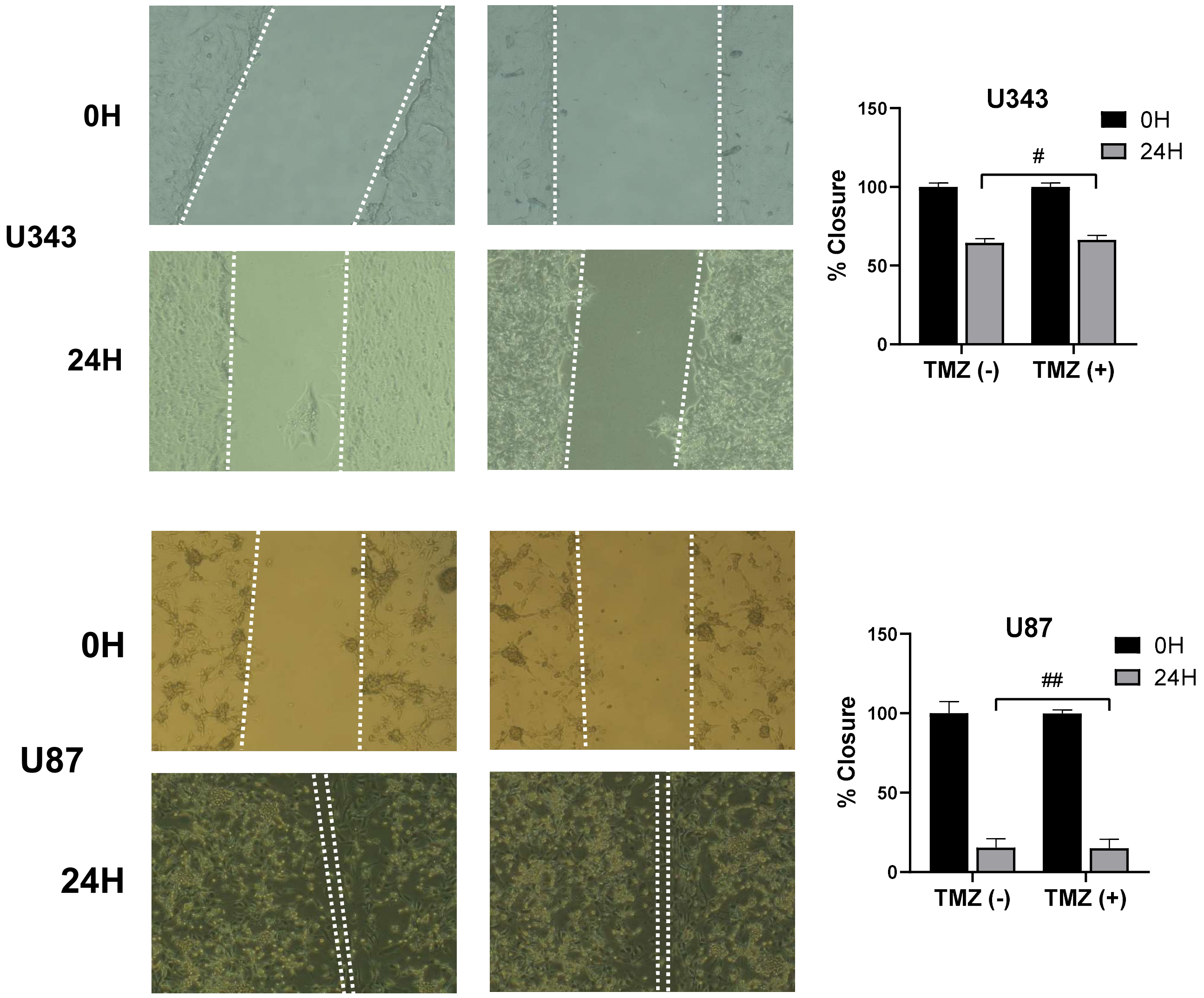

B

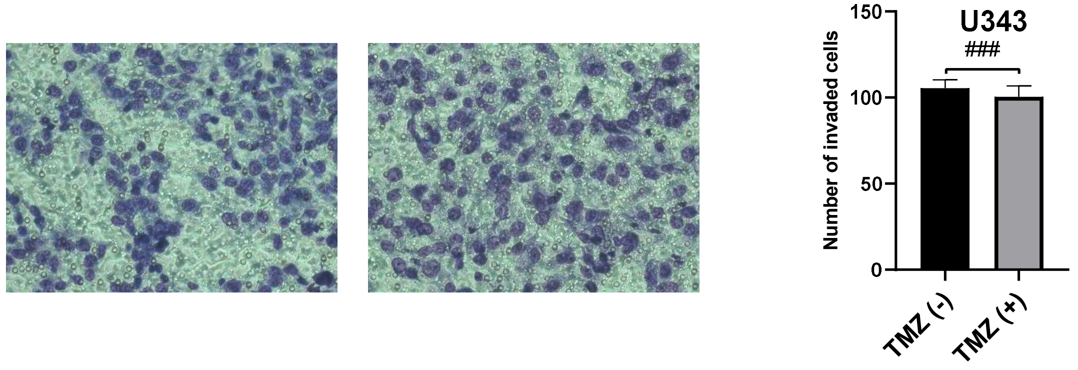

Figure S7. TMZ effects in *in vitro* migration and invasion. Scratch assay was used to compare the migration ability of GBM cell exposed with TMZ after 24 hours (A). Transwell membrane assay was used to compare invasiveness of TMZ treated GBM cells using Corning® BioCoat™ Growth Factor Reduced Matrigel Invasion Chamber (B). Each bar represents the mean ± S.D. The difference between two groups was determined by paired two-tailed Student's t-test. #p=0.3350, ##p=0.9703, ###p=0.6444.

Table S1

	Antibody	Catalog number	Company
1	β-actin	26628-22-8	Sigma-Aldrich
2	MMP9	AB19016	Merck Millipore
3	MMP9	sc-13520	Santa Cruz Biotechnology
4	MMP2	NB200-114	Novus Biologicals
5	p-p38	05-1059	Sigma-Aldrich
6	p38	sc-535	Santa Cruz Biotechnology
7	p-JNK	07-175	Sigma-Aldrich
8	JNK	3708	Cell Signaling Technology
9	p-ERK	05-797R	Sigma-Aldrich
10	ERK	sc-271269	Santa Cruz Biotechnology
11	p-AKT	05-669	Sigma-Aldrich
12	AKT	4691	Cell Signaling Technology
13	p-p65	sc-33020	Santa Cruz Biotechnology
14	p65	sc-8008	Santa Cruz Biotechnology
15	p-c-Fos	sc-81485	Santa Cruz Biotechnology
16	c-Fos	sc-166940	Santa Cruz Biotechnology
17	p-c-Jun	sc-822	Santa Cruz Biotechnology
18	c-Jun	sc-74543	Santa Cruz Biotechnology
19	TIMP-1	sc-365905	Santa Cruz Biotechnology
20	TIMP-2	sc-21735	Santa Cruz Biotechnology
21	uPA	sc-59727	Santa Cruz Biotechnology
22	uPAR	sc-376494	Santa Cruz Biotechnology
