# Supplementary material for: Temozolomide promotes matrix metalloproteinase 9 expression through p38 MAPK and JNK pathways in glioblastoma cells
Source: Sci Rep. 2024 Jun 21;14:14341. doi: 10.1038/s41598-024-65398-2 (PMC11192740; doi:10.1038/s41598-024-65398-2)
Supplement: Supplementary file 2 — Supplementary Information 2. [file 41598_2024_65398_MOESM2_ESM.pdf]

Figure 1B

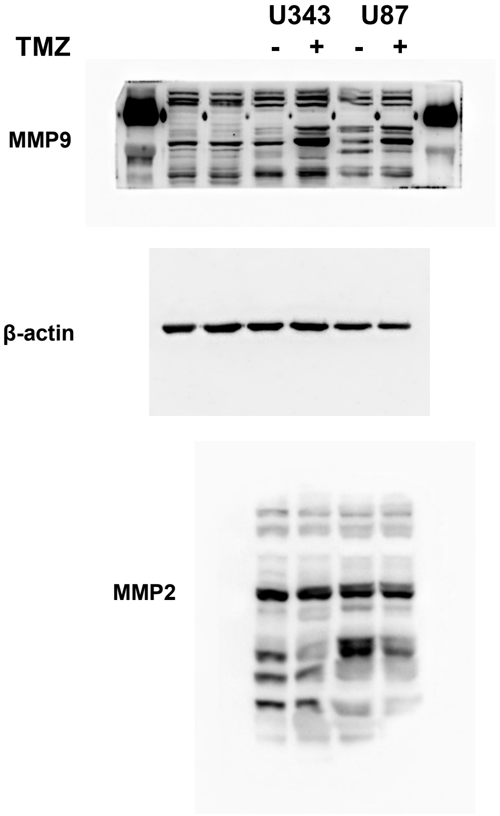

Figure 1C

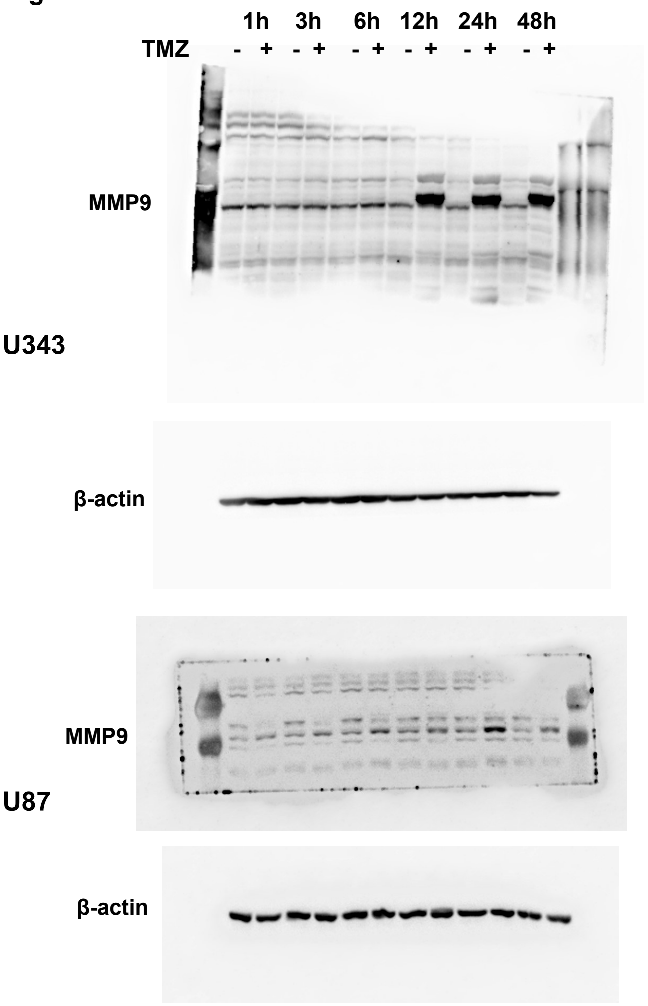

Figure 2C

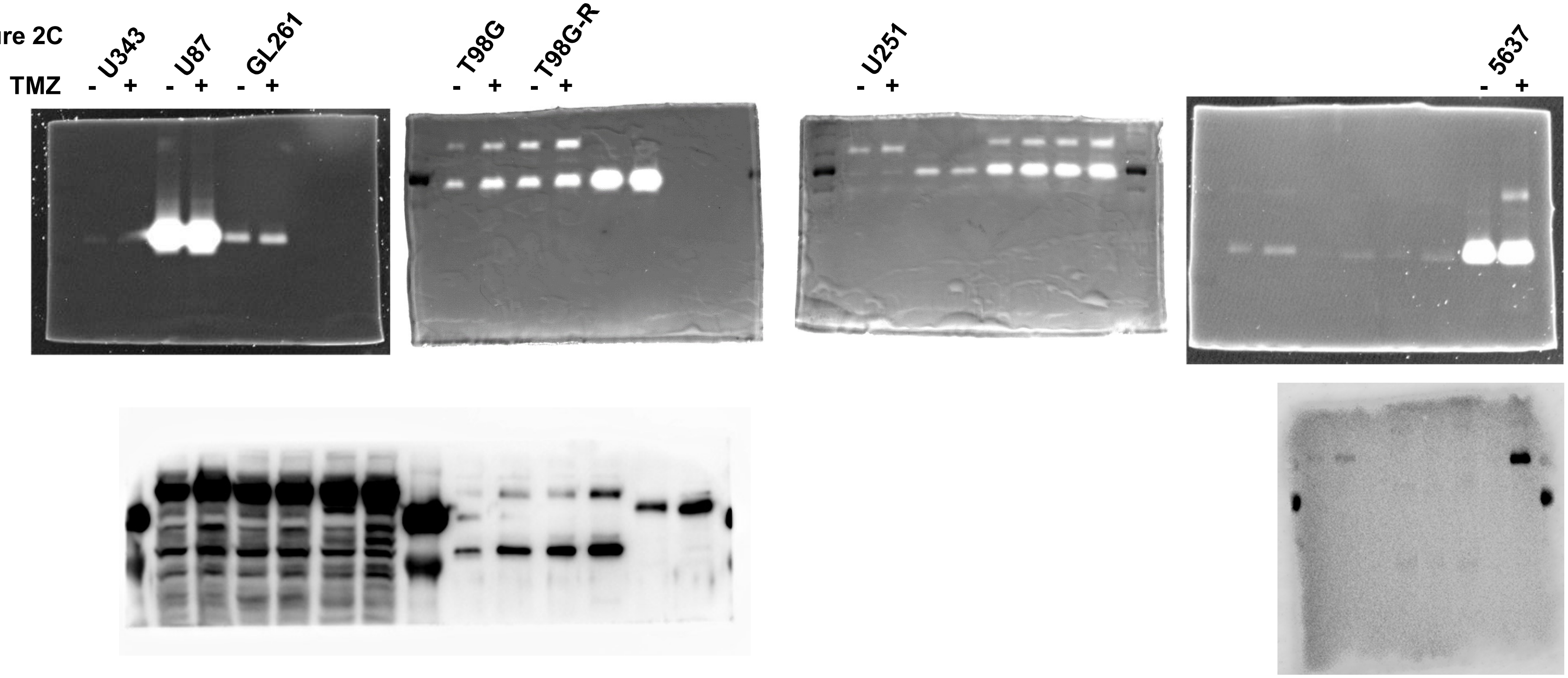

Figure 3A

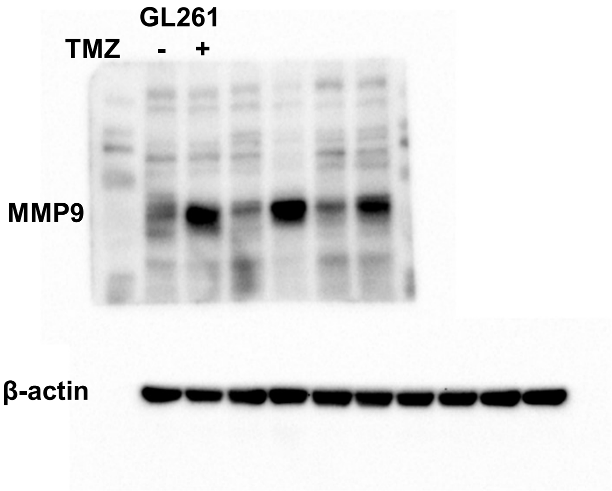

Figure 3C

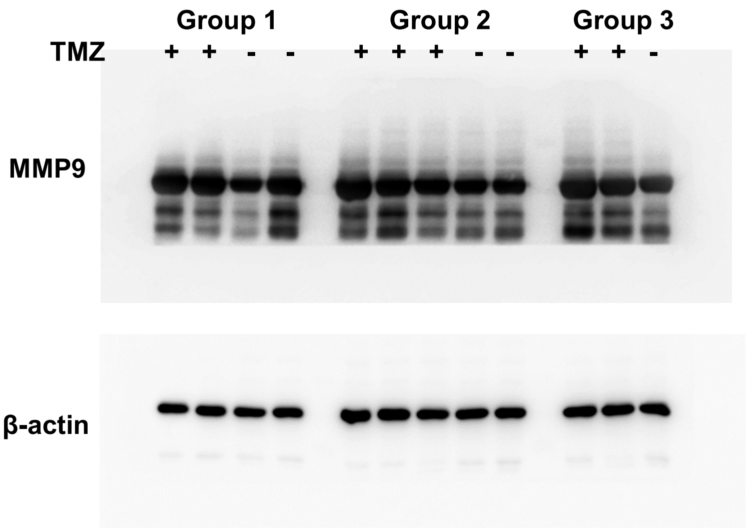

Figure 3E

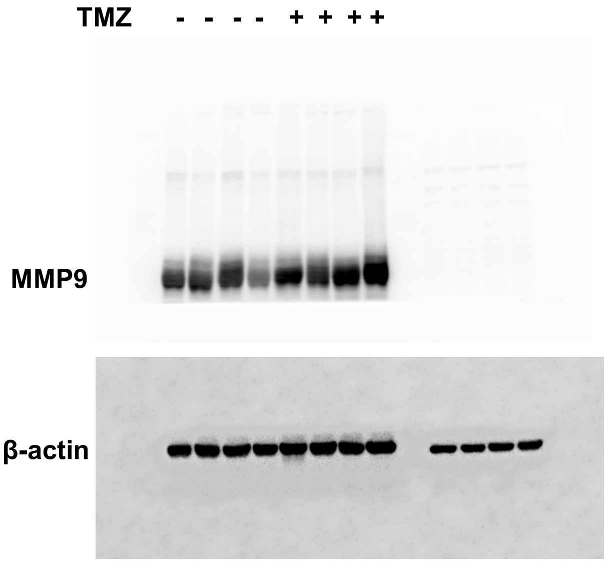

Figure 4A

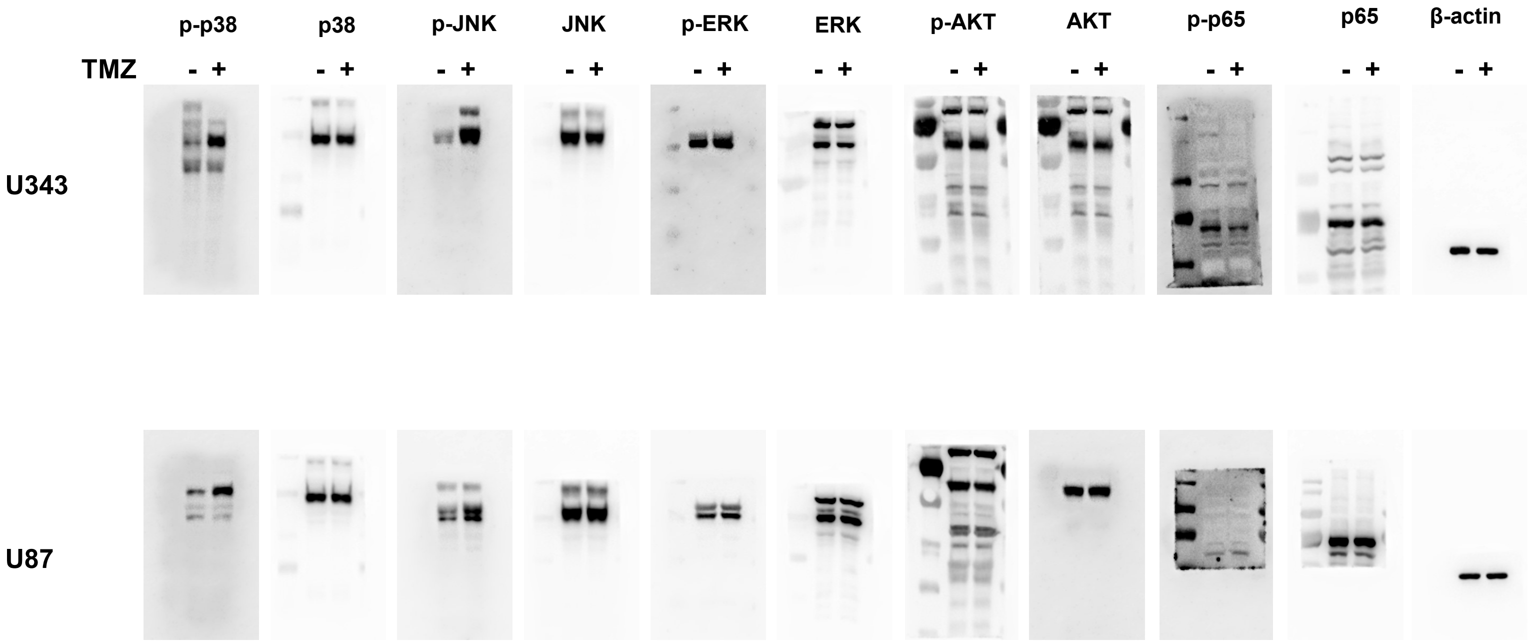

p-p38
p38
p-JNK
JNK
p-ERK
ERK
p-AKT
AKT
p-p65
p65
β-actin
TMZ
- +
U343
U87

Figure 4B

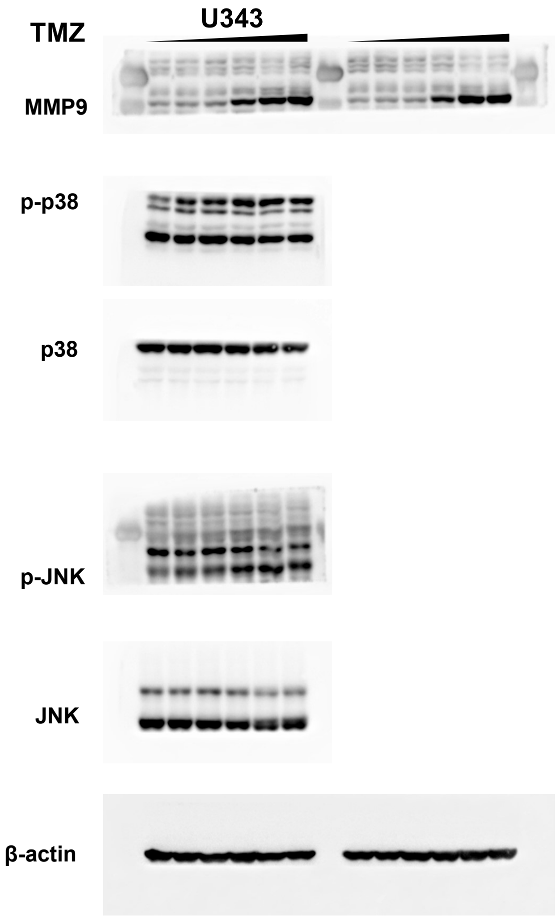

Figure 4C

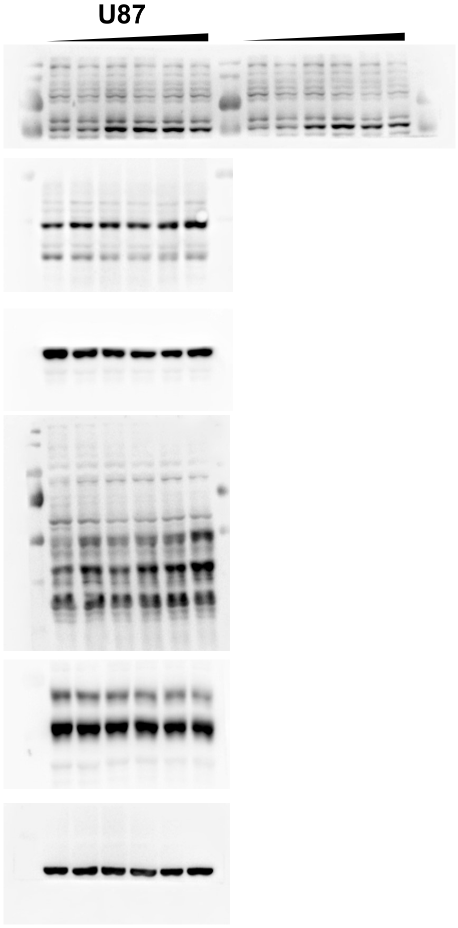

Figure 4D

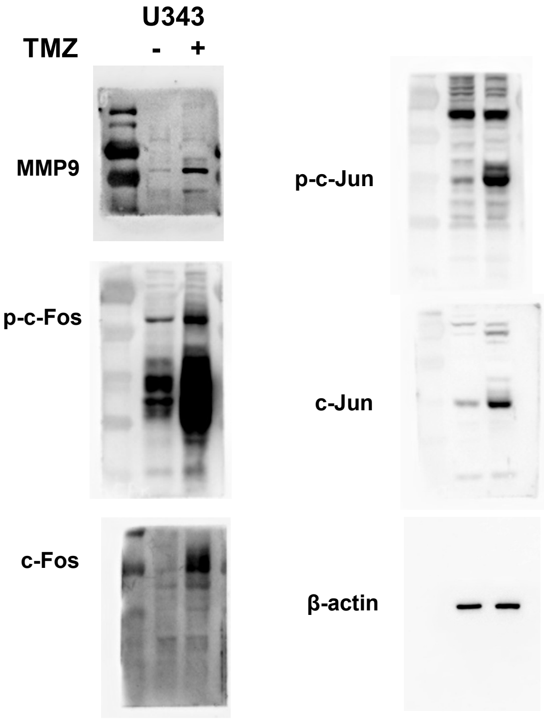

Figure 5A

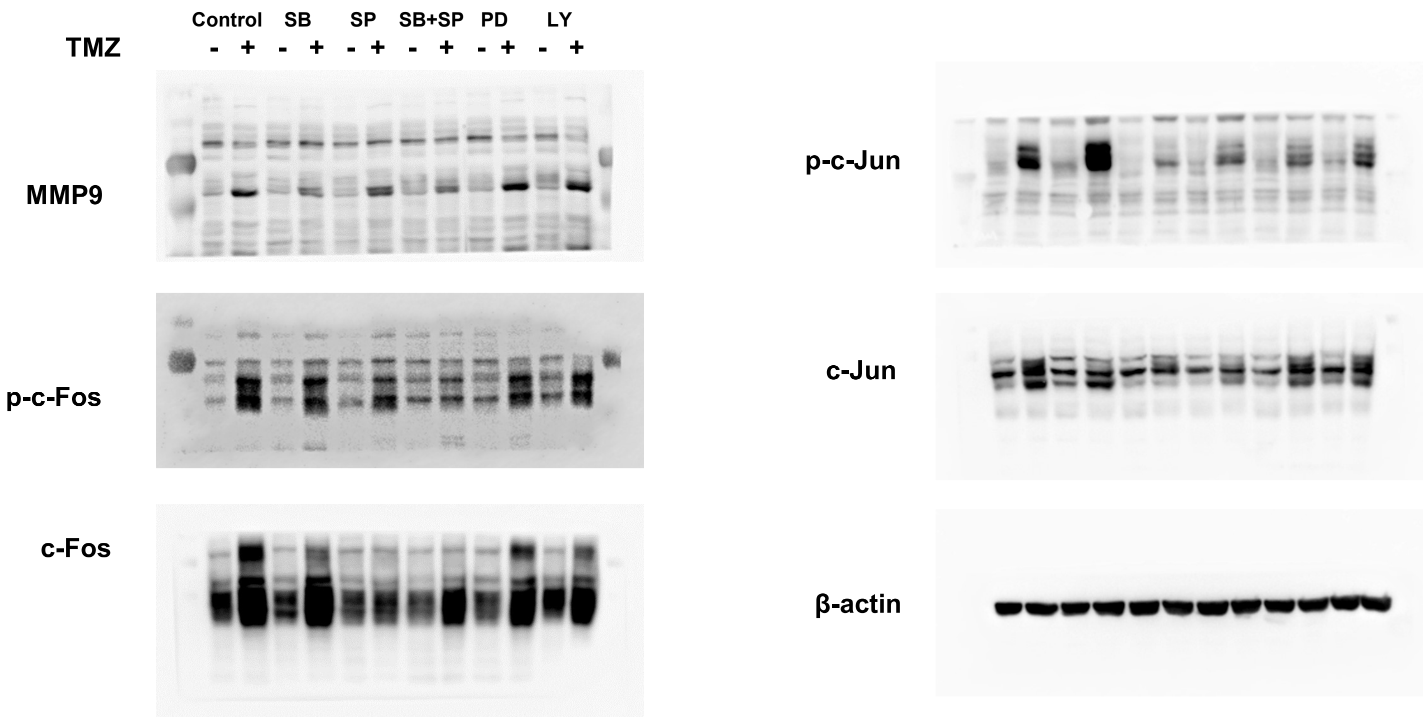

Control
SB
SP
SB+SP
PD
LY
TMZ
- + - + - + - + - + - +
MMP9
p-c-Fos
c-Fos
p-c-Jun
c-Jun
β-actin

Figure 6A

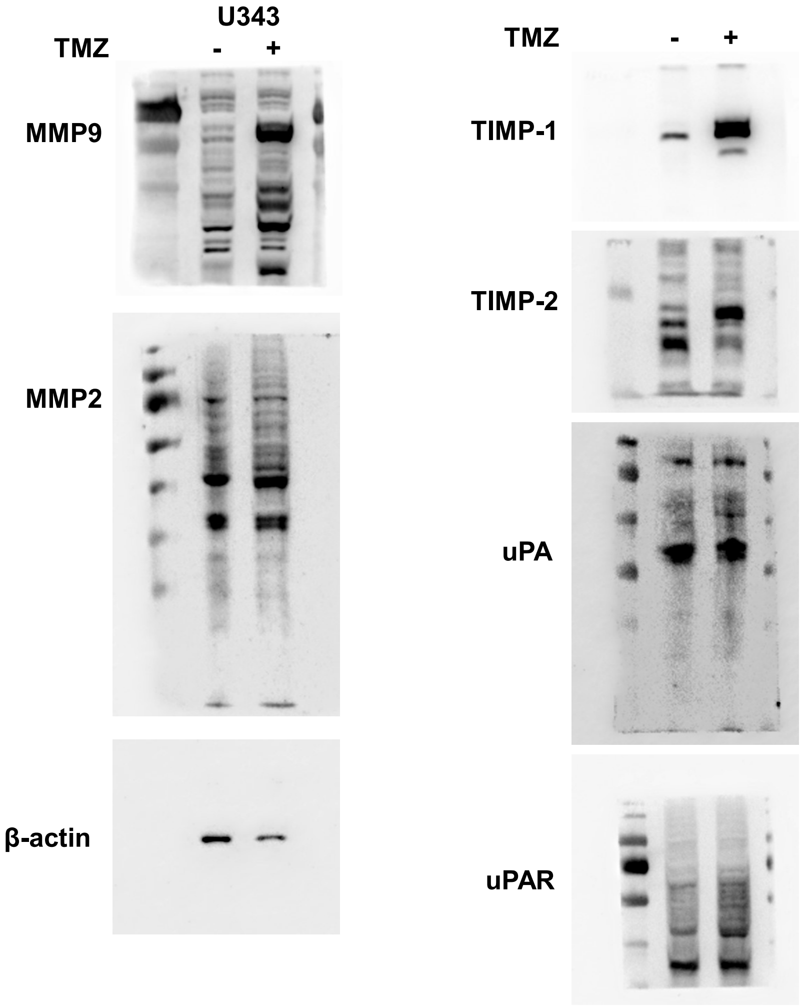

Depending on protein size, the blots were cut prior to hybridization with antibodies.

Figure S2

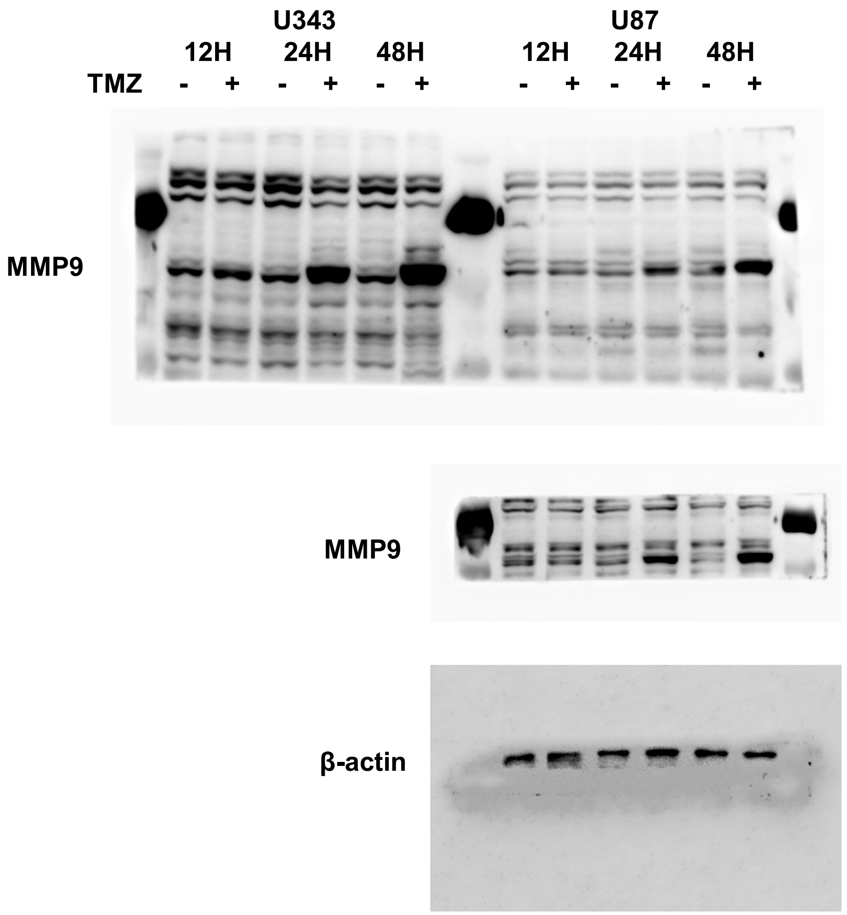

Depending on protein size, the blots were cut prior to hybridization with antibodies.

Figure S3

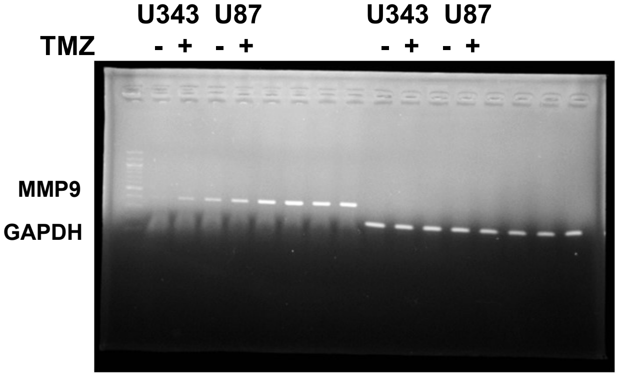

Figure S4

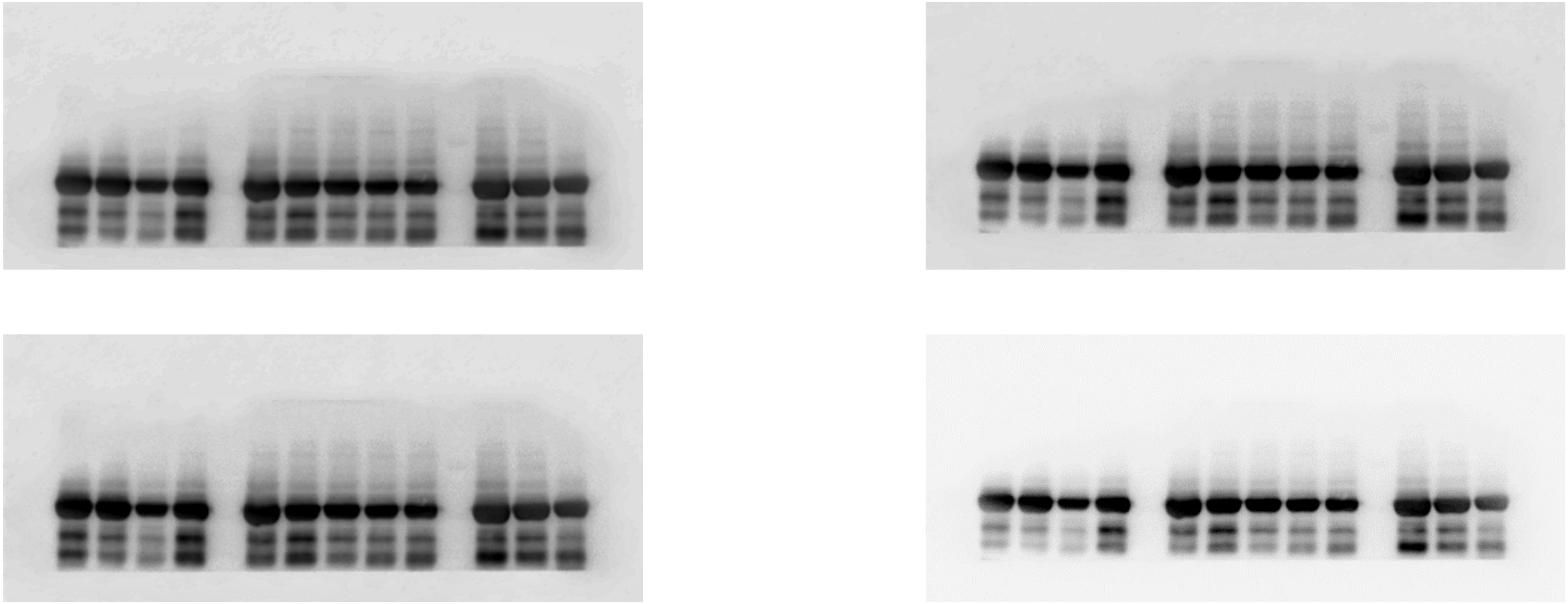

Depending on protein size, the blots were cut prior to hybridization with antibodies.

Figure S6

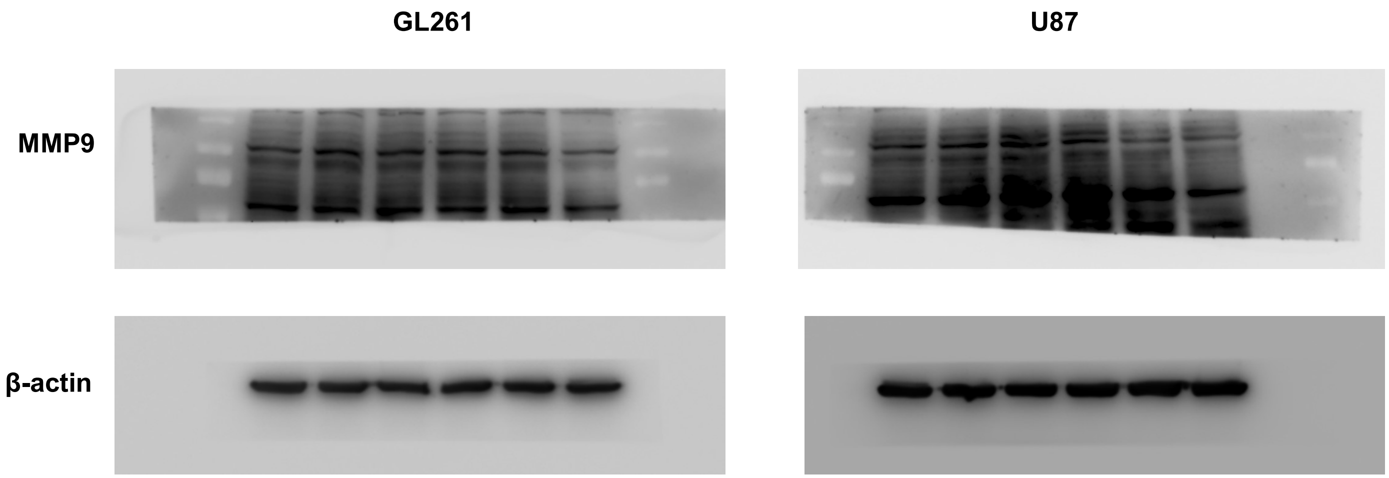

Depending on protein size, the blots were cut prior to hybridization with antibodies.
